# Polyphenol Contents, Gas Chromatography-Mass Spectrometry (GC–;MS) and Antibacterial Activity of Methanol Extract and Fractions of *Sonneratia Caseolaris* Fruits from Ben Tre Province in Vietnam

**DOI:** 10.4014/jmb.2304.04019

**Published:** 2023-09-30

**Authors:** Thien khanh Tran, Pham Thi Thu Ha, Robert. J. Henry, Dang Nguyen Thao Nguyen, Phung Thi Tuyen, Nguyen Thanh Liem

**Affiliations:** 1 Chemical Engineering in Advanced Materials and Renewable Energy Research Group, School of Technology, Van Lang University, Ho Chi Minh City 700000, Vietnam; 2 Faculty of Applied Technology, School of Technology, Van Lang University, Ho Chi Minh City, 700000, Vietnam; 3 Queensland Alliance for Agriculture and Food Innovation, The University of Queensland, St Lucia, QLD 4072, Australia; 4 High Agricultural Technology Research Institute for Mekong Delta, 94955, Vietnam (HATRI); 5 Faculty of Applied Sciences, Ton Duc Thang University, Ho Chi Minh City, 700000, Vietnam; 6 Department of Forest Plant, Faculty of Forest Resources and Environmental Management, Vietnam National University of Forestry, Hanoi, 13417, Vietnam; 7 Faculty of Natural Sciences, Quynhon University, Quynhon, Binhdinh 590000, Vietnam

**Keywords:** Antibacterial, GC–MS, flavonoid, phenolic, *Sonneratia caseolaris*

## Abstract

Plants contain a large number of phytochemical components, many of which are known as bioactive compounds and responsible for the expression of various pharmacological activities. The extract of *Sonneratia caseolaris* fruit collected in Vietnam was investigated for its total phenolic and total flavonoid contents using methanol solvent and different fractions of *S. caseolaris* fruits (hexane, ethyl acetate, n-butanol, and aqueous). GC–MS analysis was conducted to identify the bioactive chemical constituents occurring in the active extract. Further, the antibacterial activity was tested in vitro on bacterial isolates, namely *Escherichia coli*, *Staphylococcus aureus*, and *Bacillus subtilis*, using the disc diffusion method on tryptic soya agar (TSA) medium. The methanol extract showed high total flavonoid (82.3 ± 0.41 mg QE/g extract) and phenolic (41.0 ± 0.34 mg GAE/g extract) content. GC–MS of the methanol extract and different fractions of *S. caseolaris* fruits detected 20 compounds, principally fatty alcohols, fatty acids, phenols, lipids, terpenes derivatives, and carboxylic acids derivatives. A 50 mg/ml concentration of methanol extract had the strongest antibacterial activity on *E. coli*, *S. aureus*, and *B. subtilis*. Furthermore, ethyl acetate, aqueous, and n-butanol fractions inhibited *S. aureus* and *B. subtilis* the most. The results of the present study suggested that the fruits of *S. caseolaris* are rich sources of phenolic compounds that can contribute to safe and cost-effective treatments.

## Introduction

*Sonneratia caseolaris* is one of the main plants of some mangrove forests, found in less salty areas in the mangroves, usually along tidal channels with slow-flowing water that is deeply muddy, but this species is not found in coral reefs [1]. Currently, many countries have wild *S. caseolaris*, including Africa, Sri Lanka, Myanmar, Thailand, Vietnam, Cambodia, Philippines, Indonesia, Timor, Hainan Island (China), Northeast Australia, and some countries in Oceania such as Niughnia, New Guinea, Solomon Islands, New Hebrides [2]. In Vietnam, *S. caseolaris* has grown wild and is grown in coastal mangrove forests from the North to the South where there is a lot of mud and mudflats. In the North, *S. caseolaris* grows in coastal and estuary forests such as in Hai Phong, Nghe An, and Ha Tinh [3]. In the South, *S. caseolaris* is a major component of the natural coastal mangroves and grows densely along the canals of the Mekong Delta of Vietnam, the central coastal region [4]. They have great value in forestry production, and coastal protection and support coastal fisheries [5]. In extreme conditions, this is also a plant species with higher biological ecological, and physiological adaptability than other plants in the same ecosystem [6]. In some folk medicine documents, *S. caseolaris* is indicated as a valuable source of medicinal herbs. Furthermore, its sour-tasting young berry fruits are edible and used as medicine in poultices for relieving sprains [7]. This plant has been discovered to produce protective bioactive phytochemicals, making it a promising source for extracting such compounds [8] such as gallic acid as well as flavonoids such as luteolin and luteolin-7-O-glucoside [9]. It includes the compounds alkaloid, tannin, flavonoid, saponin, phytosterol, and carbohydrate [10]. Extracts of mangrove leaves have shown promise as a potential natural antibiotic source due to their high levels of trace phenolic compounds, including phenolic acids and flavonoid derivatives [11, 12].

Kasote *et al*. [13] plants have an inherent ability to synthesize antioxidants, primarily in the form of polyphenols, vitamin E, and vitamin C, as a means of safeguarding themselves against UV radiation and pathogens. The most common are phenolic acids, flavonoids, lignans, stilbenes, and tannins [14]. According to da Silva *et al*. [15] the difference in flavonoid fraction is most likely caused by the different distribution and types of phenolic compounds that are found in different fruit sections and different plant species. In addition, differences in agricultural techniques, soil nutrients, weather, fruit maturity level, and biotic and abiotic factors influence the phenolic content of fruits [16].

In previous research by Koohsari *et al*. [17], the sensitivity of gram-positive bacteria like *B. subtilis* and *S. aureus* and one gram-negative bacteria, *E. coli*, were selected to study plant extracts. *B. subtilis* is one of the few genera of bacteria that can survive in the soil, the gastrointestinal tract of ruminants, and the gastrointestinal tract of humans, making it one of the many diverse species of bacillus that can develop endospores, ropiness, sticky and stringy stability, and other characteristics due to the production of long-chain polysaccharides by the organisms [18]. There have been studies on the chemical composition and biological activity of *S. caseolaris* to confirm its antibacterial, anticancer, and antioxidant properties [19]. Besides that, *S. caseolaris* extract is considered a potential plant extract for use against pathogenic bacteria. Yompakdee *et al*. [20] reported that antibacterial activity was found in methanol extract samples from different parts, such as leaves, flowers, and fruit, of the Crabapple Mangrove tree in Thai Lan. The bark tissue of *S. caseolaris* showed antibacterial activity against *B. subtilis* and *Proteus vulgaris*, according to Simlai *et al*. [19]. In addition, it was found that the methanol extract of *S. caseolaris* fruit might inhibit the growth of microbes such as *E. coli*, *S. aureus*, and *Candida albicans* [21]. Furthermore, differences in natural conditions, such as temperature and edaphic parameters, between Vietnam and the countries mentioned above may affect chemical constituents, thereby affecting the biological activities of this species. Thus, this study aims to determine the total phenolic, total flavonoid contents and bacterial activity of methanol extract and its fractions of *S. caseolaris* fruit collected from Ben Tre Province of Vietnam. We also used GC–MS analysis to determine the chemical composition of the methanol extract and its fractions from the fruit of *S. caseolaris*.

## Materials and Methods

### Bacterial Species and Chemical Reagents

To determine the antibacterial activity of methanol extract and different fractions of *S. caseolaris* fruit, bacterial strains such as *E. coli* (ATCC 25922), *S. aureus* (ATCC 29247), and *B. subtilis* (ATCC 6633) were used in this experiment. The following chemicals were used in this study including methanol (XiLong, China), hexane (C_6_H_14_; Vietnam), ethyl acetate (C4H8O2, Vietnam), n-Butanol (C_4_H_8_O_2_, China), Amoxicillin (Vietnam), Gallic acid (C_7_H_6_O_5_; China), Folin-Ciocalteu reagent (Germany), Sodium carbonate (Na_2_CO_3_; Germany), Quercetin (C_15_H_10_O_7_; Sigma-Aldrich, Singapore), Aluminium chloride (AlCl_3_; China), Tryptic Soy Agar medium (Sigma-Aldrich, Germany).

### Plant Material and Preparation of Extract

**Fig. 1:**
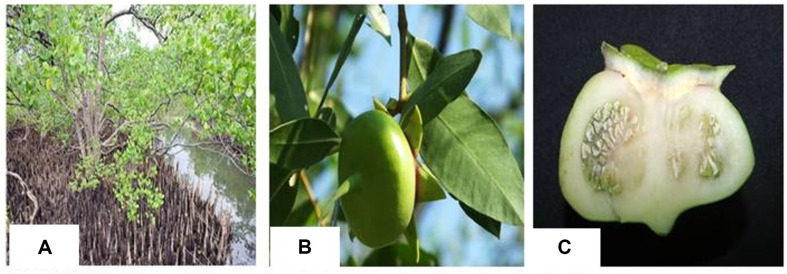
*Sonneratia caseolaris* in Ben Tre province. (**A**) *Sonneratia caseolaris* tree (**B**) fruit and (**C**) fruit in cross-section.

**Fig. 2:**
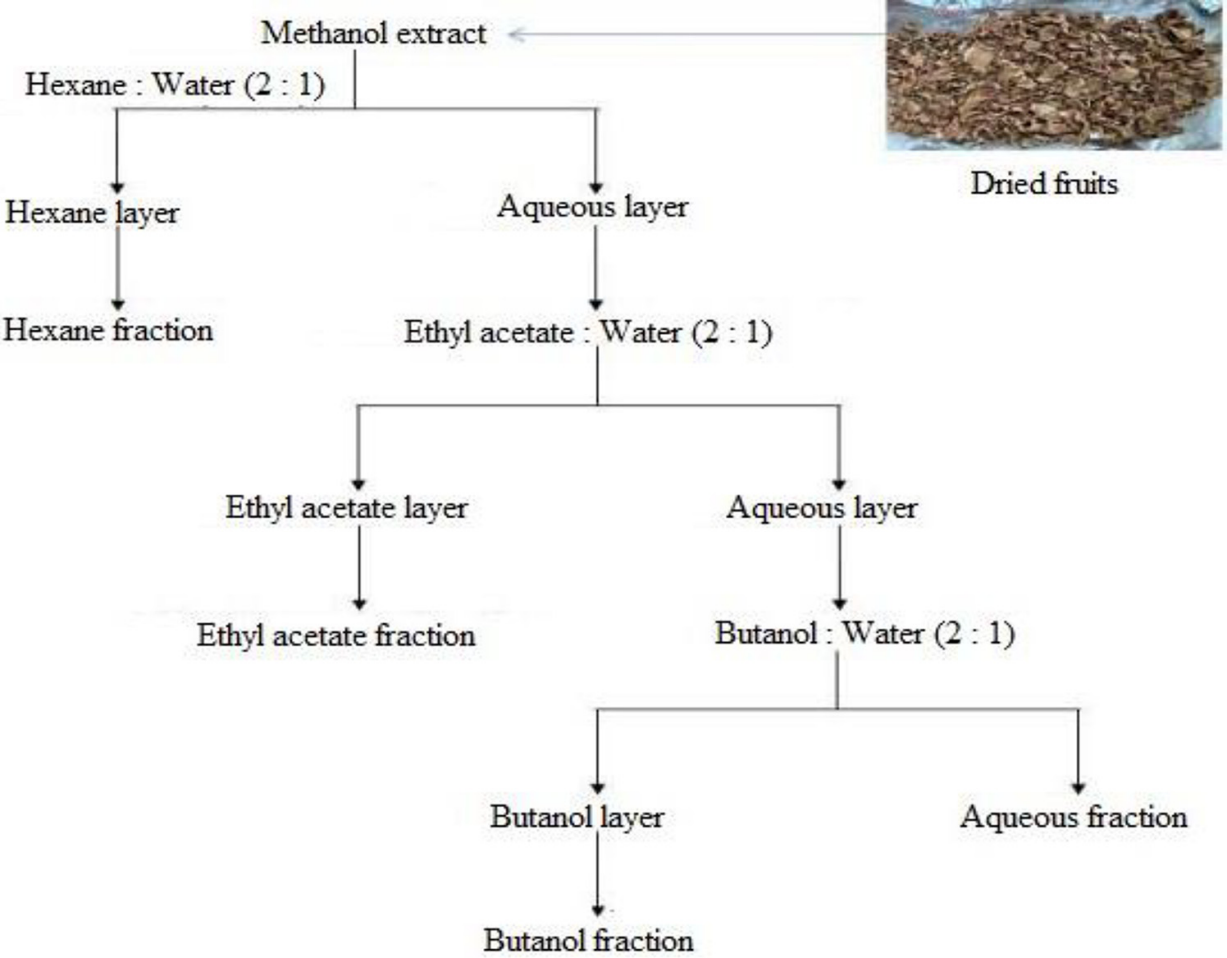
The procedure for collecting methanol extract and 4 fractions of *Sonneratia caseolaris* fruits.

The selected mangrove apple (*S. caseolaris*) fruits were collected at the bank of Ben Tre River, Ben Tre province in September 2019 (Fig. 1). Fruits were transported to the laboratory, washed, cut into slices, and dried in the drying oven at 55°C for three days. The dried fruits were ground into a fine powder. The powder of fruits was stored in a tightly closed bag and extracted by maceration. The powder (100 g) was shaken in a glass bottle containing a total of 1000 ml methanol solvent for 3 days (27–29°C). The plant extracts were filtered with a vacuum filtration apparatus and then the solvent was removed using a Rotary evaporator (SB-350-EYALA, Japan) at 30°C. The weight volume of dried material was recorded before storage at 4 – 6C until fraction. To separate polar and non-polar organic compounds in the methanol extracts of the sample, we used four solvents, including hexane, ethyl acetate, n-butanol, and distilled water as shown in Fig. 2. 200 ml of various solvents, including hexane, ethyl acetate, n-butanol, and distilled water, were dissolved in the dried extract (v/v: 2:1), yielding separate fractions at the end of the operation. All fractions were evaporated to dryness in a rotary evaporator under a vacuum at 30°C before being redissolved in methanol, hexane, ethyl acetate, n-butanol, and distilled water for further analysis.

### Estimation of Total Phenolic Content

The total phenolic content of five fractions was determined by the Folin–Ciocalteu reagent [22] with slight modifications. In summary, the reaction mixture contained 1 ml of the fraction (1 mg/ml) or standard gallic acid solution (20, 40, 60, 80, and 100 g/ml) was mixed with 2.5 ml of Folin–Ciocalteu 10%, shaken well and held for 5 min and then 2 ml of Na_2_CO_3_ (7.5 %) was added. The mixture was kept at room temperature for 60 min. The absorbance at 765 nm was read against a blank sample. Using a spectrophotometer (HACH DR/4000U, USA), the total phenolic content was determined based on a gallic acid calibration curve. The results were expressed in terms of gallic acid equivalents (mg of GAE/g extract). The samples were analysed in triplicate.

### Estimation of Total Flavonoid Content

The total flavonoid content of the five fractions was analysed according to Abdeslam *et al*. [23], with some modifications. Briefly, the reaction mixture containing 2 ml of the fraction (1 mg/ml in methanol) was mixed with 2ml of 2 % AlCl_3_ in methanol. After keeping it at room temperature for 40 min, the absorbance against a blank was read at 415 nm. The total flavonoid content was determined using a standard curve with quercetin (QE) as the standard (20, 40, 60, 80, and 100 g/ml). Total flavonoid contents were expressed as quercetin equivalent (mg QE/g extract). The samples were analysed in triplicate.

### Gas Chromatography-Mass Spectrometry (GC–MS) Analysis of the Fractions

GC–MS analysis of the five fractions was carried out using a Perkin-Elmer GC Clarus 500 system gas chromatograph interfaced with a mass spectrometer (JMS-T100 GCV, Jeol Ltd., Japan) equipped with a DB-5MS column (30 mm × 250 μm × 0.25 μm; Agilent, USA) as described by Jenecius *et al*. [24] with some modifications. 100 mg of each fraction was diluted with 1 ml of MeOH, filtered with a 0.45 μm filter, and then 1 μl was injected into a GC–MS. An electron ionization system with an ionizing energy of 70 eV was used for GC–MS detection. Helium gas (99.999 %) was used as the carrier gas at a flow rate of 1 ml/min and an injection volume of 0.5 μl was employed (ratio of 10:1). The oven temperature program was as follows: 50°C (for 5 min), with an increase of 5°C/min, to 200°C, then 10°C/min to 280°C, ending with isothermal at 280°C. Analysis of the mass spectrum from GC–MS was processed using the database of the National Institute of Standards and Technology (NIST). After obtaining the spectrum of the unknown component, it was compared to the spectra of known components that were archived in the NIST library.

### Antibacterial Activity Test

The disc diffusion method for antibacterial activity was applied according to Razmavar *et al*. [25] with some modifications. 100 ml of each bacterial suspension was uniformly spread on the tryptone soya agar medium in a Petri dish. The five fractions were diluted to concentrations of 10, 30, and 50mg/ml. Three sterile paper discs with a diameter of 6 mm are placed on the surface of each agar plate and then impregnated with 30 ml of diluted fractions. The positive and negative controls were amoxicillin and methanol, respectively. Plates were incubated for 24 h at room temperature (25 – 30°C). Antibacterial activity was assessed by measuring the inhibition zone diameter around the discs. The test was performed three times. If the inhibition zone is ³ 15 mm in size, the inhibitory response is categorised as strong (+++). The inhibitory response is categorised as medium (++) for an inhibition zone of 10–15 mm in size, weak (+) for 9 mm, and no resistance (-) for 0–6 mm.

### Data Analysis

The experimental data were expressed as mean ± standard deviation (SD). The statistical significance of the means inhibition zone data of the fractions for each bacterium was performed with a two-way ANOVA followed by Tukey's post hoc multiple comparison tests to determine the significant differences at *p* < 0.05. Statistical analyses used the statistical analysis software (SAS) (version 8.2).

## Results

### Total Phenolic and Flavonoid Contents

The total phenolic content in the methanol extract and different fractions of *S. caseolaris* estimated by the Folin–Ciocalteu method using gallic acid as the standard is shown in Table 1. The total phenolic content ranged from 59.6 to 82.7 mg GAE/g extract. The highest total phenolic content was found in the butanol and methanol fractions with 82.7 ± 0.81 mg GAE/g extract and 82.3 ± 0.41 mg GAE/g extract, respectively. The total phenolic content of the hexane extract from the fruit was significantly lower (*p* < 0.05) when compared with the other fractions. The content of total flavonoids ranged from 1.81 ± 0.24 mg QE/g extract for aqueous to 41.0 ± 0.34 mg QE/g extract for methanol extract (Table 1). The highest total flavonoid content of *S. caseolaris* fruits was found in the methanol extract.

### Chemical Profiles Identified by GC–MS

**Fig. 3:**
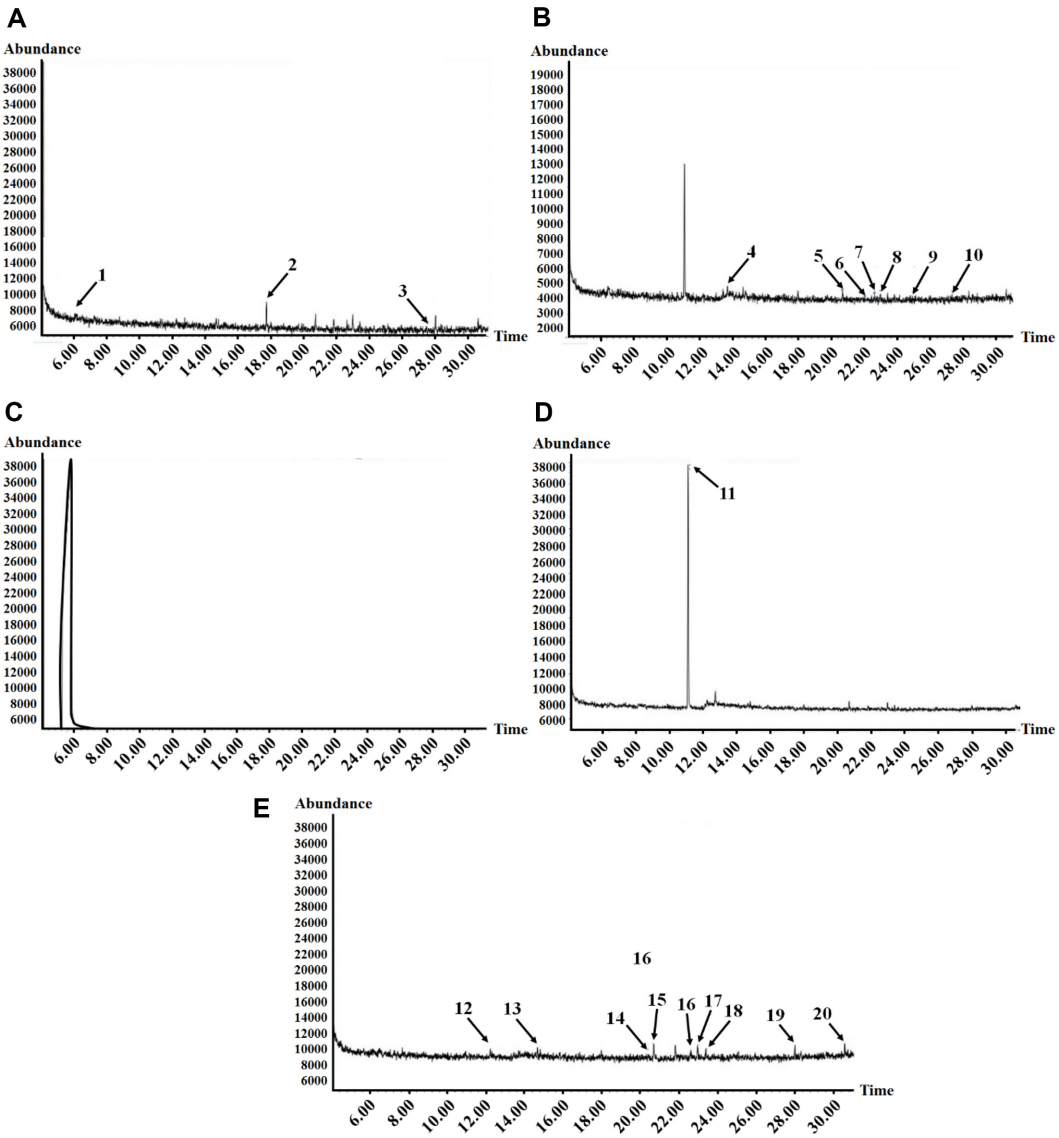
GC-MS total ion chromatogram of S.caseolaris fruits methanolic extract and fractions. Peak identification: (**A**-Methanol extract: 1, 13-Heptadecyn-1-ol; 2, Estragole; 3, 2-Hexadecanol), (**B**-Hexane fraction: 4, 1-Octanol; 5, Myristynoyl pantetheine; 6, Triacetin; 7, 2-Myristynoyl pantetheine; 8, Cubedol; (9) Cyclobarbital; (10) β-curcumene); (CEthyl acetate: (11) Butanoic acid); (D-n-Butanol: (11) Butanoic acid); (E-Aqueous: (12) α-Santonin; (13) 2-Myristynoyl pantetheine; (14) Tridecanedial; (15) Falcarinol; (16) Prednisone; (17) Safrole; (18) tert-Hexadecanethiol; (19) Rhodopin; (20) Geldanamycin).

The chemical components in the methanol extract and different fractions of *S. caseolaris* fruit were determined using GC–MS analysis (Table 2, Fig. 3).

GC–MS analysis of *S. caseolaris* extract showed total components in methanol extract, hexane, ethyl acetate, n-butanol, and aqueous fractions were 3, 7, 1,1 and 9 respectively (Table 2). Twenty compounds were detected by using GC–MS, principally belonging to fatty alcohols, fatty acids, phenols, lipids, terpenes derivatives, and carboxylic acid derivatives. The aqueous fraction was found to contain the highest number of components followed by the hexane fraction. The same component by only butanoic acid was detected in ethyl acetate and butanol fractions (Table 2). The solvents that brought the best results to extract phytochemicals in *S. caseolaris* fruits were hexane and aqueous, fractions while methanol, ethyl acetate, and butanol showed the least efficacy.

### Antibacterial Activity Test

**Fig. 4:**
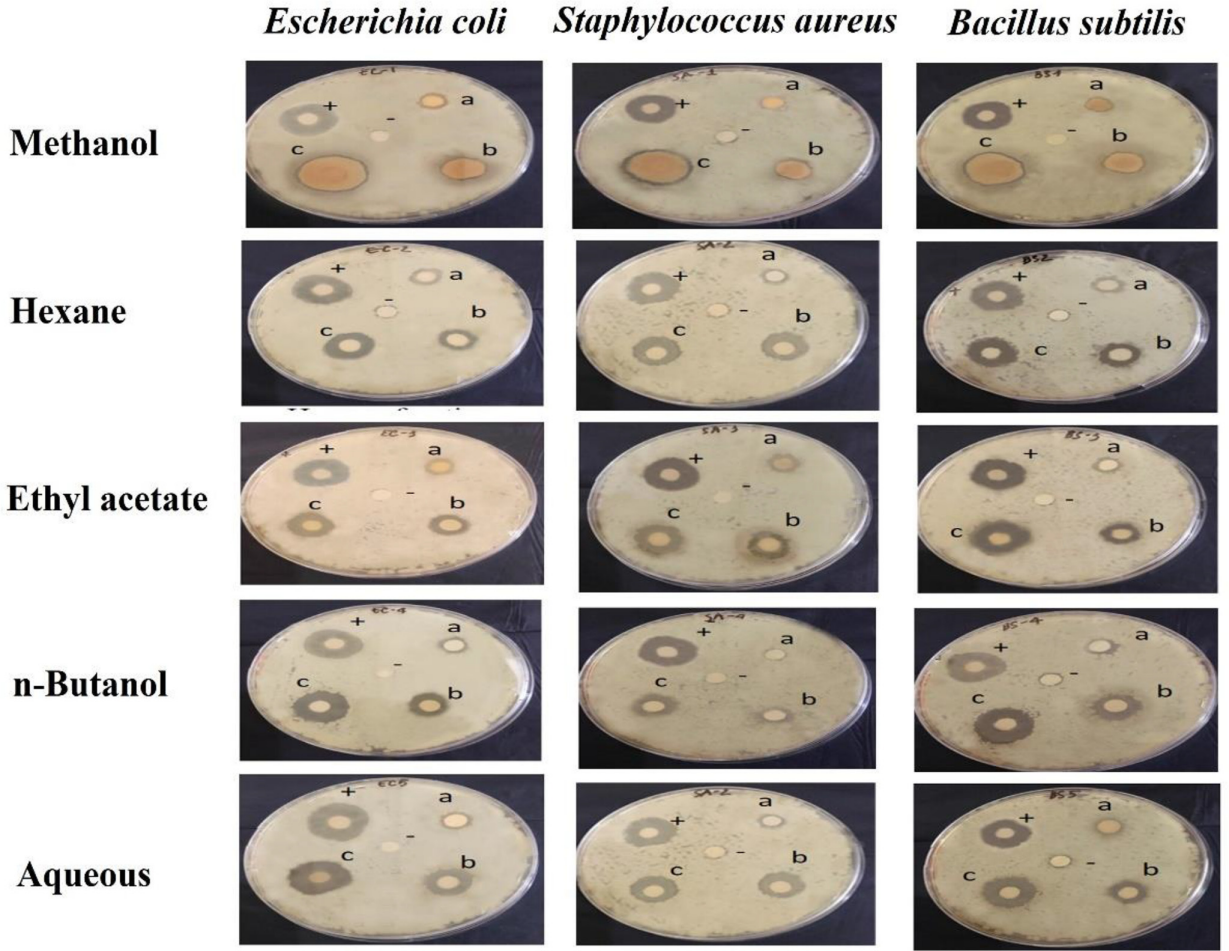
Test of *Escherichia coli*, *Staphylococcus aureus*, and *Bacillus subtilis* resistance activity of methanol extract and 4 fractions of *Sonneratia caseolaris* fruits. (+) positive control amoxicillin 50 mg/ml; (-): negative control methanol; (a): 10 mg/ml; (b): 30 mg/ml; (c): 50 mg/ml.

The antibacterial activity of the methanolic extract and different fractions against *E. coli*, *S. aureus* and *B. subtilis* are shown in Table 3 and Fig. 4. The results showed that the methanol extract and different fractions of 10 mg/ml, 30 mg/ml, and 50 mg/ml all had inhibitory effects on *E. coli*, *S. aureus* and *B. subtilis* at different levels. Most concentrations tested 50 mg/ml showed the strongest antibacterial capacity with the largest zone of inhibition. When the bacterial activity of individual fractions of fruits of *S. caseolaris* was measured the methanol extract exhibited the strongest inhibitory activity against *E. coli* with a mean zone of inhibition of 14.23 mm in diameter, ethyl acetate and aqueous fractions exhibited the strongest inhibitory activity against *S. aureus* (11.56 and 11.77mm in diameter, respectively), and n-butanol fraction inhibited the growth of *B. subtilis* with the largest zone of inhibition (13.33 mm in diameter). These results indicate that the fruits of *S. caseolaris* have antibacterial activity against *E. coli*, *S. aureus*, and *B. subtilis* and that the extraction of fruits with methanol, ethyl acetate, n-butanol and aqueous solvent might be utilised in the treatment of infectious diseases caused by resistant microbes.

## Discussion

Plants contain a large number of phytochemical components, many of which are known as bioactive compounds and responsible for the expression of various pharmacological activities [26]. There was higher than those found in *S. ovata* fruit with 22.5 mg GAE/g and Cashew apple fruit with 53 mg GAE/g, according to Wetwitayaklung *et al*. [27] and Silva *et al*. [28] respectively. *S. apetala*, a plant of the same genus was examined for leaves, stem bark and roots and extracts were shown to have phenolics 47.5 ± 2.22 mg GAE/g, 42.7 ± 2.75 mg GAE/g, and 42.8 ± 1.67 mg GAE/g, respectively, according to Banerjee *et al*. [29]. In this study, methanol, the most polar extract, was found to contain the highest content of total phenolic (82.3 mg/g) and flavonoid (41.0 mg/g) as compared to other fractions (Table 2). Previous studies have demonstrated that phenolic compounds have shown potential biological activities such as antioxidant, antidiabetic, hepatoprotective, anti-inflammatory, antimicrobial, and anticancer [30, 31]. In this study, the total flavonoid content of methanol extract for *S. caseolaris* fruit was higher than that of Hossain and Rahman [32] and Liu *et al*. [33] who reported those in Bangladeshi pineapple fruit and mangrove plants (*S. apetala* Buch) extracts. Another study reported that two flavonoids, namely luteolin and luteolin 7-O-βglucoside were found in *S. caseolaris* fruit, which explains the antioxidant activity of the fruit [34]. The fruits of *S. caseolaris* were rich in phenolics and it can be proposed that the biological activity of this species could be due to the presence of flavonoids and other phenolics. Methanol was found to facilitate the extraction of more phytochemical compounds due to being more polar [35].

GC–MS is one of the most exact methods to identify secondary metabolites in plant extracts with the help of the NIST library. The current result of the GC–MS analysis of *S. caseolaris* extract showed the presence of several important chemical compounds like fatty alcohols, fatty acids, phenols, lipids, terpenes derivatives, and carboxylic acids derivatives. A study by Bandaranayake [36] reported that chemical compounds such as phenols, terpenes, and carboxylic acid derivatives found in mangroves have been used and are in demand in industry and modern medicine. McGaw and Staden [37] noted that fatty acids are important constituents of plants and are commonly known to possess antimicrobial activities. In this study, a total of chemical compounds belonging to fatty alcohols, fatty acids, phenols, lipids, terpenes derivatives, and carboxylic.

The extract concentration for antibacterial assay in the present study was determined as 10, 30 and 50 mg/ml. Saif [38] in a study evaluated the antimicrobial activity of methanol extract from *Elaeophorbia drupifera* (Thonn.) Stapf. (Euphorbiaceae) at a concentration of 50 mg/ml against *S. aureus*. In the extract concentration of 50 mg/ml that was applied in the Yavuz *et al*. [49] study, antibacterial activity was observed against the *E. coli* using the methanol extract of some plant species belonging to the Lamiaceae family (*Stachys annua*, *Scutellaria salviifolia*, and *Nepeta nuda*). Similar to the results of our study evaluated the antimicrobial activity of methanol extract and different fractions of leaf basil against *E. coli* set as 10, 30, and 50 mg/ml [40]. The methanol extract and different fractions of S caseolaris fruits had relatively high antibacterial activity against *E. coli*, *S. aureus*, and *B. subtilis*. The antibacterial effects of the methanolic fruit extracts of *S. caseolaris* against *S. aureus*, *E. coli*, and *C. albicans* were reported by Ahmad *et al*. [21]. In this study, compared with *S. caseolaris* fractions of the same concentration, there was not much difference, and all fractions gave very weak levels shown by the zone of inhibition value (< 8 mm). Also, differences in the ability of Mangrove fruit extracts to inhibit or kill the growth of microbes may be caused by sensitivity to antimicrobial compounds contained in extracts, wherein the constituent is more sensitive to the yeast and the gram-negative bacteria compared to the gram-positive bacteria. In the previous study, Simlai *et al*.[19] also reported that the methanol and water extracts from the bark tissue of *S. caseolaris* exhibited antibacterial activity against *B. subtilis* and *E. coli* with 18.3 ± 0.76 mm and 15.8 ± 0.29 mm, respectively. The antibacterial activity of Sonneratia was assessed by three different agar-based assays with methanol extract from seeds and gallic acid for testing by Jongjan *et al*. [41]. They showed that methanol extract was able to inhibit *S. aureus* and *C. albicans* but did not inhibit *E. coli* while gallic acid only showed activity against *S. aureus*. The study by da Costa *et al*. [42] showed the methanolic extract of the bark of *S. caseolaris* has been found to possess the highest activity against *B. subtilis*. In this research, among solvents used to extract, the best activity was methanol at 50 mg/ml which exhibited antibacterial activity against *E. coli*, *S. aureus*, and *B. subtilis*. Additionally, ethyl acetate, aqueous and n-butanol fractions showed the strongest inhibitory activity with *S. aureus* and *B. subtilis*. Therefore, conducting extensive research is necessary for the isolation, purification, and standardization of the active antibacterial components in *S. caseolaris* fruits depending on the GC–MS results of each segment.

The results of GC–MS and preliminary photochemical testing indicated that *S. caseolaris* fruits contained numerous bioactive phytoconstituents belonging to fatty alcohols, fatty acids, phenols, lipids, terpenes derivatives, and carboxylic acid derivatives that may be responsible for antibacterial activity. 1- Dodecanol compound belongs to fatty alcohol with a long chain registered for *S. aureus* with the highest antibacterial activity by Togashi *et al*.[43]. According to Marwa *et al*. [44], 2-Hexadecanol, which was discovered through GC–MS analysis of *Paecilomyces lilacinus* acetone extract, exhibited antimicrobial and antibacterial activity. There have been several reports on the antibacterial activities of estragole against the *S. aureus* 1199B strain [45, 46], *S. aureus* RN4220 and *S. aureus* RN4220 [47]. Minqing *et al*. [48] isolated twenty-four compounds from Chinese *S. caseolaris* stem and twigs, but none of the compounds showed significant antibacterial activity against *S. aureus*. In the present study, three compounds (13-Heptadecyn-1-ol, Estragole and 2-Hexadecanol) were identified in GC–MS analysis of the methanol extract of the fruit *S. caseolaris* which was evaluated as the highest antibacterial activity. There have been several reports on the antibacterial activities of long-chain fatty alcohols [49-51]. In this study, we found a 13-Heptadecyl^-1^-ol compound of long-chain fatty alcohol in the methanol extract of the fruit *S. caseolaris* that showed potent antibacterial activity using GC–MS. This study provides the basis for further extensive research in exploring the possibility of new naturally biologically active compounds with antibacterial activity.

## Figures and Tables

**Table 1:** Total phenolic and flavonoid content of *Sonneratia caseolaris* fruit fractions.

Fractions	Total phenolic content (mg GAE/g)	Total flavonoid content (mg QE/g)
Methanol	82.27 ± 0.41^a^	40.95 ± 0.34^a^
Hexane	59.58 ± 2.70^d^	16.54 ± 0.44^c^
Ethyl acetate	77.67 ± 0.32^b^	26.28 ± 0.93^b^
n-Butanol	82.67 ± 0.81^a^	9.13 ± 0.34^d^
Aqueous	70.26 ± 0.35^c^	1.81 ± 0.24^e^

Mean ± Standard deviation; in the same column, means with the same letter were not significantly different (*p* < 0.05).

**Table 2:** Components detected in fractions of *Sonneratia caseolaris* fruit by GC-MS.

Fractions	No.	Retention time	Name of the putative compound	Peak area	Chemical class	Mass (g/mol)
Methanol	1	5.097	13-Heptadecyn-1-ol	74.01	Long-chain fatty alcohol	252.44
	2	17.707	Estragole	586.26	Phenol	148.20
	3	27.998	2-Hexadecanol	1225.51	Fatty alcohol	242.44
Hexane	4	13.697	1-Octanol	4731.81	Fatty alcohol	130.23
	5	20.666	Myristynoyl pantetheine	704.36	Carboxylic acid	554.72
	6	21.836	Triacetin	2635.65	Glycerolipids	218.20
	7	22.672	2-Myristynoyl pantetheine	587.09	Carboxylic acid	554.72
	8	22.991	Cubedol	658.25	Prenol lipid	222.37
	9	25.127	Cyclobarbital	449.96	Barbituric acid	236.27
	10	27.618	β-curcumene	3006.88	Sesquiterpene	204.35
Ethyl acetate	11	11.095	Butanoic acid	42605.00	Fatty acid	88.106
n-Butanol	11	11.103	Butanoic acid	11158.00	Fatty acid	88.106
Aqueous	12	12.226	α-Santonin	151.42	Terpene	246.30
	13	14.662	2-Myristynoyl pantetheine	286.68	Carboxylic acid	554.72
	14	20.713	Tridecanedial	500.33	Volatile oil	212.33
	15	21.789	Falcarinol	535.55	Fatty alcohol	244.37
	16	22.627	Prednisone	33.56	Steroid	358.43
	17	22.967	Safrole	342.14	Colorless oil	162.19
	18	23.394	tert-Hexadecanethiol	236.03	Colorless liquid	258.51
	19	27.998	Rhodopin	766.47	Carotenoid	554.89
	20	32.744	Geldanamycin	138.80	Phenol	560.64

**Table 3:** The diameter of the zone (mm) of inhibition against *Escherichia coli*, *Staphylococcus aureus*, and *Bacillus subtilis* by the methanol extract and different fractions from *Sonneratia caseolaris* fruit at different concentrations.

Bacterial strains	Fractions	Concentrations						Mean of Concentration
		10 mg/ml	Level	30 mg/ml	Level	50 mg/ml	Level	
*Escherichia coli*	Methanol	7.67 ± 0.58^f^	+	14.00 ± 0.00^c^	++	21.00 ± 1.00^a^	+++	14.22 A
	Hexane	7.33 ± 0.58^f^	+	11.67 ± 0.58^de^	++	14.67 ± 0.58^c^	++	11.22 C
	Ethyl acetate	7.33 ± 0.58^f^	+	11.33 ± 0.58^e^	++	13.67 ± 0.58^cd^	++	10.78 C
	n-Butanol	7.00 ± 0.00^f^	+	14.33 ± 0.58^c^	++	17.67 ± 0.47^b^	+++	12.99 B
	Aqueous	7.33 ± 0.58^f^	+	14.67 ± 0.58^c^	++	17.33 ± 1.53^b^	+++	13.11 B
	Mean of Concentration	7.33 C		13.20 B		16.86 A		
*Staphylococcus aureus*	Methanol	0.00^f^	-	11.67 ± 0.58^d^	++	21.00 ± 1.00^a^	+++	10.890 AB
	Hexane	7.33 ± 0.58^e^	+	11.67 ± 0.58^d^	++	12.67 ± 0.58^cd^	++	10.5567 B
	Ethyl acetate	7.67 ± 0.58^e^	+	12.33 ± 1.15^d^	++	14.67 ± 0.58^bc^	++	11.5567 A
	n-Butanol	0.00^f^	-	8.33 ± 0.58^e^	+	11.33 ± 1.15^d^	++	6.5533 C
	Aqueous	7.33 ± 0.58^e^	+	11.67 ± 0.58^d^	++	16.33 ± 0.58^b^	+++	11.7767 A
	Mean of Concentration	4.47 C		11.13 B		15.20 A		
*Bacillus subtilis*	Methanol	7.00 ± 0.00^f^	+	11.33 ± 0.58^de^	++	18.67 ± 0.58^a^	+++	12.3333 B
	Hexane	7.67 ± 0.58^f^	+	10.33 ± 0.58^e^	++	16.33 ± 0.58^b^	+++	11.4433 C
	Ethyl acetate	7.33 ± 0.58^f^	+	12.33 ± 0.58^cd^	++	17.33 ± 0.58^ab^	+++	12.3300 B
	n-Butanol	8.33 ± 0.58^f^	+	13.33 ± 1.52^c^	++	18.33 ± 0.58^a^	+++	13.3300 A
	Aqueous	0.00^g^	-	12.33 ± 0.58^cd^	++	16.67 ± 0.58^b^	+++	9.6667 D
	Mean of Concentration	6.07 C		11.93 B		17.47 A		
P (Fractions)	< 0.05							
P (Concentration)	< 0.05							
P (Fractions * Concentration)	< 0.05							

Mean ± SD; in the same row, means with the same letter were not significantly different (P < 0.05). Levels as (+++): Strong; (++): Moderate; (+): Weak; (−): Negative
